# The Therapeutic Potential of Brown Adipocytes in Humans

**DOI:** 10.3389/fendo.2015.00156

**Published:** 2015-10-13

**Authors:** Craig Porter, Maria Chondronikola, Labros S. Sidossis

**Affiliations:** ^1^Metabolism Unit, Shriners Hospitals for Children-Galveston, Galveston, TX, USA; ^2^Department of Surgery, University of Texas Medical Branch, Galveston, TX, USA; ^3^Department of Preventive Medicine and Community Health, University of Texas Medical Branch, Galveston, TX, USA; ^4^Department of Nutrition and Dietetics, Harokopio University of Athens, Athens, Greece; ^5^Department of Internal Medicine, University of Texas Medical Branch, Galveston, TX, USA

**Keywords:** adipose tissue, mitochondria, uncoupling protein 1, thermogenesis, obesity

## Abstract

Obesity and its metabolic consequences represent a significant clinical problem. From a thermodynamic standpoint, obesity results from a discord in energy intake and expenditure. To date, lifestyle interventions based on reducing energy intake and/or increasing energy expenditure have proved ineffective in the prevention and/or treatment of obesity, owing to poor long-term adherence to such interventions. Thus, an effective strategy to prevent or correct obesity is currently lacking. As the combustion engines of our cells, mitochondria play a critical role in energy expenditure. At a whole-body level, approximately 80% of mitochondrial membrane potential generated by fuel oxidation is used to produce ATP, and the remaining 20% is lost through heat-producing uncoupling reactions. The coupling of mitochondrial respiration to ATP production represents an important component in whole-body energy expenditure. Brown adipose tissue (BAT) is densely populated with mitochondria containing the inner mitochondrial proton carrier uncoupling protein 1 (UCP1). UCP1 uncouples oxidative phosphorylation, meaning that mitochondrial membrane potential is dissipated as heat. The recent rediscovery of BAT depots in adult humans has rekindled scientific interest in the manipulation of mitochondrial uncoupling reactions as a means to increase metabolic rate, thereby counteracting obesity and its associated metabolic phenotype. In this article, we discuss the evidence for the role BAT plays in metabolic rate and glucose and lipid metabolism in humans and the potential for UCP1 recruitment in the white adipose tissue of humans. While the future holds much promise for a therapeutic role of UCP1 expressing adipocytes in human energy metabolism, particularly in the context of obesity, tissue-specific strategies that activate or recruit UCP1 in human adipocytes represent an obligatory translational step for this early promise to be realized.

## Introduction

Obesity and its associated metabolic complications (hyperlipidemia, insulin resistance, and glucose intolerance) have become significantly more prevalent in the United States and around the world in recent years (1, 2). One third of Americans are either obese (1) or exhibit symptoms of pre-diabetes (3). Worldwide, excessive adiposity is responsible for more than three million deaths and is a significant cause of disability (4). Increasing adiposity is associated with derangements in glucose and lipid metabolism and in the development of insulin resistance (5, 6). Therapeutic strategies aimed at preventing obesity or treating its metabolic complications typically target dietary intake and/or physical activity, but to date do not seem to exhibit a great deal of long-term efficacy. Mitochondrial proton leaks, i.e., respiration uncoupled from ATP production, accounts for around 20% of total mitochondrial respiration (7). The physiological role ascribed to this uncoupling of oxidative phosphorylation is heat production (thermogenesis). While thermogenesis represents a significant portion (~20%) of whole-body energy expenditure (7, 8), its therapeutic role in energy metabolism has only recently begun to receive attention.

**Figure 1:**
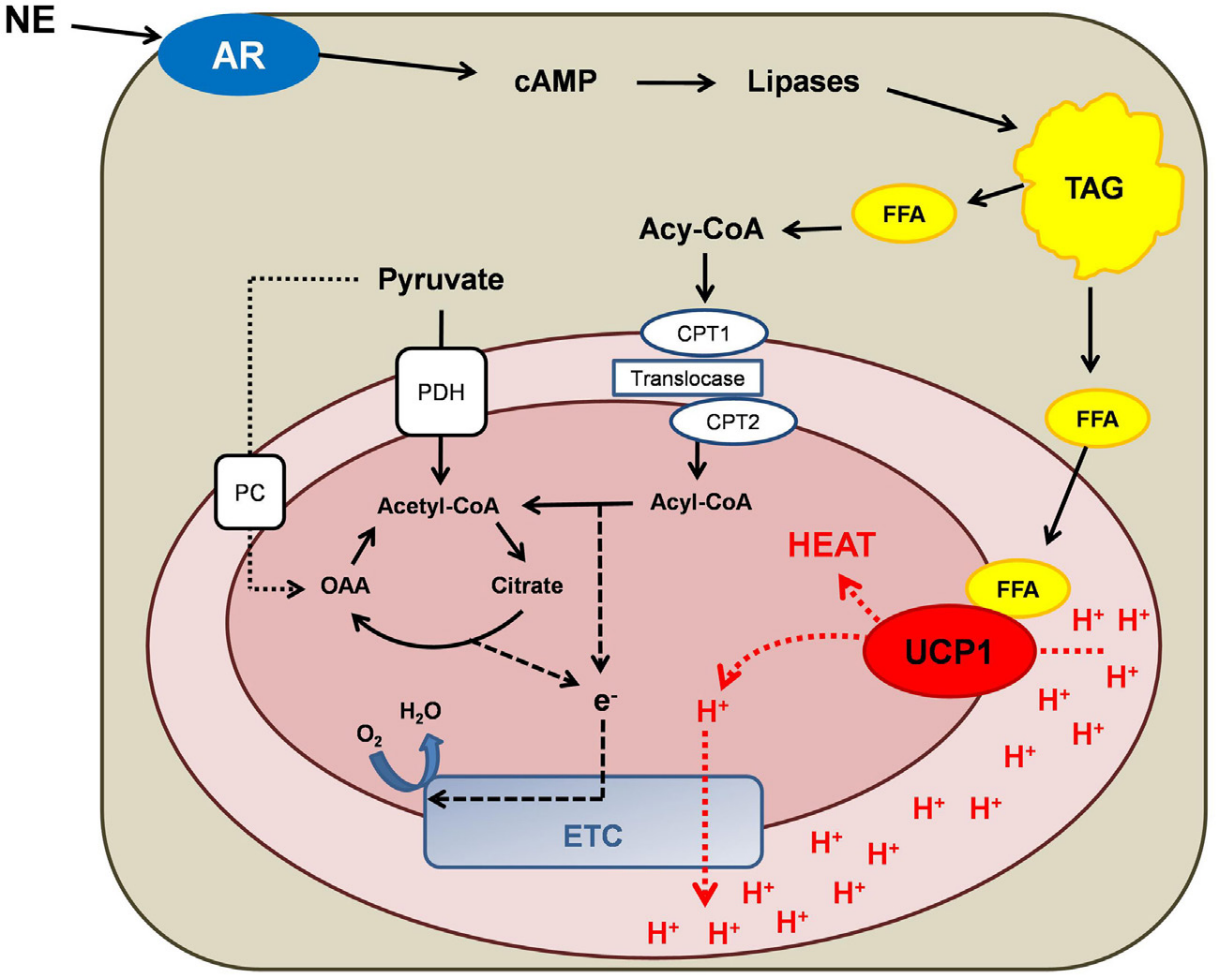
**Schematic overview of mitochondrial energy transduction within a brown adipocyte mitochondrion**. Norepinephrine (NE) activation of adrenergic receptors (AR) causes an increase in cytosolic cyclic adenosine monophosphate (cAMP) levels. The subsequent activation of lipases results in the lipolysis of triacylglycerol (TAG), which increases intracellular free fatty acid (FFA) concentrations. FFAs fulfill two principal metabolic fates: (i) FFAs bind to and activate uncoupling protein 1 (UCP1), thus switching on UCP1-mediated proton (H^+^) conductance. (ii) Activated FFAs (acyl-CoA) can be transported into the mitochondrion via the carnitine palmitoyl transferase (CPT) system and be oxidized to acetyl-CoA, thereby potentiating anaplerosis and providing reducing equivalents for the electron transport chain (ETC). Pyruvate also participates in mitochondrial anaplerosis and the production of reducing equivalent by being decarboxylated to acetyl-CoA by pyruvate dehydrogenase (PDH) or being carboxylated by pyruvate carboxylase (PC), forming oxaloacetate (OAA).

Brown adipose tissue (BAT) is the major tissue responsible for non-shivering thermogenesis in mammals (9). The recent (re)discovery of BAT in human adults (10–12) has triggered scientific interest in the role of BAT in human energy metabolism. Unique features of BAT include an abundance of mitochondria that contain the transmembrane carrier protein thermogenin, most commonly referred to as uncoupling protein 1 (UCP1) (13). Upon activation by long-chain fatty acids, UCP1 acts as an inner mitochondrial proton carrier (14), allowing protons to reenter the mitochondrial matrix independently of ATP synthase, thus uncoupling mitochondrial respiration from ATP production (9). This process alters mitochondrial energy transduction, where mitochondrial membrane potential is lost as heat (Figure 1). The purpose of this review is to summarize the current evidence regarding the role of the human adipose tissue as a therapeutic target against obesity and its related metabolic complications.

## The Role of Brown Adipose Tissue in Energy Balance and Obesity

Although the role of BAT in energy metabolism and obesity has been studied in detail in rodents (15–17), our understanding of the role BAT plays in human energy metabolism remains in its infancy. For obvious reasons, it is more difficult to manipulate and study BAT in humans. Retrospective reviews of medical records and positron emission tomography–computed tomography (PET/CT) scans, originally performed for diagnostic purposes, demonstrate an association between BAT and body mass index (BMI) (10, 18, 19) and with non-alcoholic fatty liver disease (20). The retrospective nature of these studies along with the fact that the PET/CT scans were performed under non-standardized conditions limit the generalizability of these data. However, these findings are bolstered by prospective studies which report a correlation between BAT volume and activity with BMI (11, 21), total fat mass (11, 21), and both abdominal subcutaneous and visceral adiposity (21). Moreover, weight loss via bariatric surgery has been linked to increased BAT activity (22), further suggesting that BAT may contribute to energy balance in human beings.

Acute (non-shivering) cold exposure studies have been recently performed in an attempt to estimate the contribution of BAT to cold-induced energy expenditure. However, the reported results have been highly variable. These studies reported that acute cold exposure (2–4 h at 16–19°C) induces BAT activation, resulting in a 13–27% increase in resting energy expenditure (REE) (11, 23, 24). In contrast, Ouellet et al. reported an 80% increase in REE with acute cold exposure (for 2 h at ~18°C) (25), a result over threefold higher than those of other studies, possibly attributable to muscle shivering. Muzik et al. used oxygen 15 (^15^O)-PET/CT in an attempt to more directly quantify the contribution of BAT to energy expenditure (26, 27). In contrast to previous studies, these investigators found that BAT minimally contributed to the reported increase in energy expenditure (15–25 kcal/day). Although these two studies shed doubt on the role of BAT in energy expenditure in humans, the rather short cold stimulation protocol (30 min at 18°C) may not have been adequate to fully activate BAT. Indeed, BAT recruitment via daily mild cold exposure (17°C for 6 weeks) was associated with increased thermogenesis and decreased body fat, supporting a relationship between BAT activity and adiposity (28). Similarly, Lee et al. performed a crossover cold acclimation study in five healthy participants. These researchers showed that BAT recruitment was reversibly associated with increased post-prandial energy expenditure (diet-induced thermogenesis) after cold exposure, further supporting the role of BAT in energy expenditure (29).

When interpreting the results of these studies, one important point to consider is that chronic weight gain can result from even a small discordance in daily energy balance (e.g., an energy surplus of as little as 25 kcal/day in humans can result in a weight gain of 1 kg/year). Thus, even sporadic BAT activation for limited periods during the course of the day could conceivably have a significant cumulative impact on energy balance and adiposity over several months or years.

## Can Human Brown Adipose Tissue Alter Glucose Homeostasis and Insulin Sensitivity?

**Figure 2:**
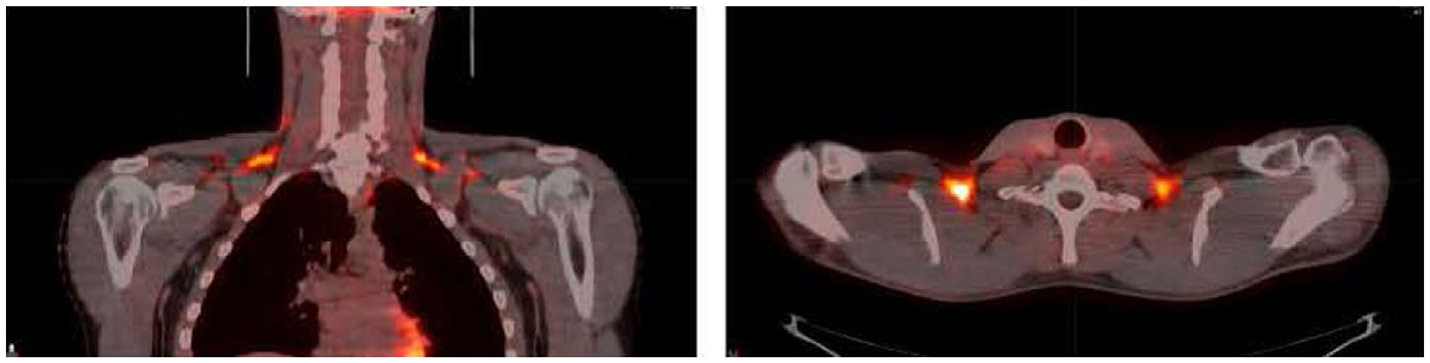
**Coronal (left) and transverse (right) 2-deoxy-2-[18F]fluoro-d-glucose (^18^F-FDG) positron emission tomography (PET)-computed tomography (CT) images from a volunteer during cold exposure**. The intense orange color in the supraclavicular area corresponds to brown adipose tissue. Image from Ref. (24). Reprinted with permission.

Brown adipose tissue has been touted as therapeutic tissue which may protect against hyperglycemia in insulin-resistant individuals. In murine models of extreme cold exposure, BAT is responsible for the majority of whole-body glucose disposal (30), while transplantation of BAT into the abdominal visceral adipose tissue improves glucose tolerance and insulin sensitivity (31). In humans, the involvement of BAT in systemic glucose metabolism is evident from ^18^F-FDG uptake images from PET/CT scans (Figure 2) performed for diagnostic purposes (32). Moreover, evidence from retrospective medical record review studies indicates an inverse relationship between BAT activity, diabetes, and glycemia (10, 19, 33, 34). Further, prospective studies have shown that acute cold exposure increases glucose uptake in BAT, where BAT glucose uptake rate per unit of tissue was higher than that of muscle (25, 35, 36), underscoring the oxidative potential of BAT. Specifically, Orava et al. reported that cold exposure resulted in a 12-fold increase in glucose disposal in BAT only, but not in other tissues (35). Similar results have been reported by Ouellet et al. (25). These data suggest that upon activation, BAT clears plasma glucose. However, Ouellet et al. demonstrated that BAT minimally contributed to whole-body plasma glucose utilization, which might be due to the short duration of cold exposure (3 h at ~18°C) and the presence of mild shivering during cold exposure (25).

The opposing results of the aforementioned studies question the physiological significance of human BAT in systemic glucose metabolism. In an attempt to address this, we studied glucose metabolism in individuals with BAT (BAT+) or without BAT (BAT−) under cold exposure and thermoneutral conditions by infusing isotopically labeled glucose in the fasted state and during a hyperinsulinemic-euglycemic clamp. We found that cold exposure increased whole-body glucose disposal in the fasted and insulin-stimulated state only in BAT+ individuals (24), suggesting that BAT can indeed play a significant role in systemic glucose metabolism in humans. Theoretically, if BAT remained chronically active, it could dispose ~23 g of glucose in 24 h [equating to approximately 8.4 kg of glucose (33,600 kcal) in a year]. Similarly, Lee et al. recently reported that increased BAT activity via cold acclimation improved post-prandial glycemia (29). Collectively, these studies support the notion that upon activation, BAT can indeed play a significant role in glucose homeostasis. Therefore, BAT may represent a novel therapeutic target in the management of hyperglycemia.

## Does Human Brown Adipose Tissue Play a Role in Systemic Lipid Metabolism?

Free fatty acids (FFAs) constitute the primary substrate for BAT (37). Moreover, FFAs bind to and activate UCP1, allowing proton transfer from the intermembrane space to the mitochondrial matrix resulting in heat production (9). Upon activation, BAT initially oxidizes intracellular lipid stores to produce heat (9). In contrast, prolonged BAT stimulation leads to increased uptake of FFA derived from circulating FFA and lipolysis of circulating lipoproteins, presumably when local lipid stores become depleted (9). BAT activation has been recently reported to increase the systemic clearance of TG-rich lipoproteins in rodents, lending credence to the notion that BAT may protect against hyperlipidemia (30, 38). Moreover, rodent BAT has been shown to release factors that increase vascular permeability in BAT, allowing TG-rich lipoproteins to enter the interstitial space (30).

The role of BAT in human lipid metabolism remains largely unexplored. Results from some (11, 35), but not all (23), cold exposure studies report a reduction in the respiratory gas exchange ratio during cold exposure, indicating increased fat oxidation in humans during BAT activation. Ouellet et al. (25) studied the role of BAT in lipid metabolism in more detail. Using PET/CT and a fatty acid tracer, ^18^F-fluoro-thiaheptadecanoic acid (^18^FTHA), they showed that 3 h of cold exposure increased BAT FFA uptake but that this accounted for <1% of the total FFA turnover (25). The conclusion of this study was that thermogenic BAT relies predominantly on the limited intracellular substrates (i.e., triglycerides stored inside BAT) to fuel mitochondrial thermogenesis (25), which questions the physiological significance of human BAT in whole-body lipid metabolism. However, the relatively short duration of the study might have been insufficient to deplete the intracellular lipid stores underestimating the contribution of BAT in the systemic lipid metabolism.

We recently studied BAT+ and BAT− individuals under mild cold exposure and thermoneutral conditions while infusing stable isotopes of glycerol and palmitate in order to directly trace lipid turnover in the fasted state and during a hyperinsulinemic-euglycemic clamp. We found that during cold exposure BAT+ individuals had greater whole-body lipolysis and FFA oxidation compared with BAT− participants, indicating that increased lipid mobilization from WAT provides FFA to sustain thermogenesis in BAT (39). Moreover, BAT+ individuals were protected from the cold-induced adipose tissue insulin resistance noted in the BAT− group (40). Our data suggest that BAT may indeed play an important role in the regulation of systemic lipid metabolism. The discord between our preliminary findings and those of others (25) may be related to the duration of cold exposure. For example, we studied individuals after ~5 h of cold exposure, while Ouellet et al. performed a 3 h cold exposure study. Whether there is a time-dependent depletion of BAT lipid stores, at which point BAT switches to using systemic FFAs as a fuel source, remains unknown.

## Can Human WAT Really *Brown*?

More than 30 years ago, Young et al. demonstrated the appearance of brown adipocytes in the parametrial fat pad of mice following acclimation to severe cold exposure (41). This was confirmed when Cousin et al. demonstrated that severe cold exposure or treatment with a β3 adrenoreceptor agonist induced brown adipocytes within WAT (42). More recently, Shabalina et al. demonstrated that acclimation to cold (~5°) results in the browning of inguinal WAT. Importantly, these researchers showed the presence of UCP1 protein while demonstrating its function (via GDP-sensitive respiration) in inguinal WAT of cold-acclimated mice (43), thus demonstrating that these mitochondria had functional UCP1. Interestingly, per milligram of tissue, inguinal WAT from cold-acclimated mice developed a thermogenic capacity ~50% of that of intrascapular BAT (43). This striking plasticity of murine subcutaneous WAT to develop a thermogenic phenotype has potential implications for humans, given that most individuals have only 100 g or less of BAT, but many kilograms of subcutaneous WAT. If WAT in humans were able to exhibit the same plasticity as in mice, this would have huge implications for energy expenditure and substrate (glucose, lipids, and potentially amino acid) metabolism.

Unlike the inguinal subcutaneous WAT depot of mice, there is no evidence to suggest that human WAT has a comparable ability to alter its thermogenic capacity in response to an environmental stress. Indeed, chronic (10 days), albeit mild cold exposure, which activates BAT, does not alter the phenotype of WAT in humans (44). This suggests that a more profound and/or chronic adrenergic stress is likely needed to induce browning in humans. Indeed, this is perhaps reasonable considering that classic rodent models of browning typically acclimate rodents to extremely cold temperatures (~5°C) for several days, if not weeks (41–43). Considering that a thermoneutral temperature for a mouse is somewhere close to 30°C, it would be reasonable to theorize that chronic exposure to a temperature 25°C below thermoneutrality would be required to induce browning of WAT in humans.

Since cold exposure protocols used in rodents cannot be replicated in humans, researchers have looked to pathologies associated with chronic adrenergic stress. Over 30 years ago, it was reported that patients with pheochromocytoma, a tumor of the adrenal medulla which results in a chronic elevation in catecholamine secretion, had BAT surrounding their kidneys (45). More recently, Frontini et al. (46) reported the existence of multilocular adipocytes that stained positive for UCP1 within the omental adipose tissue of pheochromocytoma patients. This evidence suggests that human WAT may contain brown adipocytes, but unfortunately only histological and genomic measurements were made on these WAT samples. Whether these omental adipocytes, which showed morphological similarities to that of brown adipocyte and immune-reactivity to UCP1, had functionally thermogenic mitochondria was not addressed.

Burn trauma represents a unique injury model as it is accompanied by prolonged adrenergic stress and a hypermetabolic state. To determine if subcutaneous WAT can acquire a brown fat phenotype, we collected biopsy samples of WAT from severely burned individuals. We found that when prospectively following patients after burn injury, UCP1 mRNA and UCP1 protein were induced in subcutaneous WAT at ~2 weeks post-injury (47). Moreover, this was accompanied by a similar time-dependent increase in mitochondria respiratory capacity and whole-body metabolic rate (47). Thus, it appears that human subcutaneous WAT has the ability to brown. In the context of burn trauma, this makes physiological sense, since following the destruction of their skin barrier burn victims have difficulty in conserving heat and maintaining core temperature.

To the best of our knowledge, our recent data provide the first evidence of browning of WAT in humans (47). However, we should note that while we did indeed see induction of UCP1, morphological adaptations, and increased mitochondrial respiratory in permeabilized WAT samples in a time-dependent manner after burn, the increase in thermogenic capacity (~threefold) was rather modest when compared with those seen in rodent WAT after chronic cold exposure. For example, Shabalina et al. (43) showed that the mitochondrial respiratory capacity of mitochondria isolated from the BAT of mice kept at 30°C was ~10-fold greater than that of mitochondria isolated from inguinal WAT. However, when acclimated to 5°C, the respiratory capacity of BAT mitochondria was only ~1.7-fold greater than that of inguinal WAT mitochondria. Indeed, BAT mitochondrial respiratory capacity was only marginally greater in cold-acclimated mice compared with thermoneutral mice. In contrast, WAT mitochondrial respiratory capacity was ~500% greater in inguinal WAT of cold-acclimated mice compared with thermoneutral animals. This remarkable plasticity, where murine inguinal WAT can adopt a thermogenic capacity akin to that of BAT, does not appear to be conserved in other mammals such as humans. Indeed, while we saw a threefold increase in subcutaneous WAT respiratory capacity after burn injury, respiration per milligram of tissue was still ~20-fold lower than what we have measured in human intrascapular BAT (47).

## The Therapeutic Potential of UCP1-Positive Mitochondria: Should we Activate BAT or Brown WAT?

Much emphasis has been placed on manipulating UCP1 in order to increase thermogenesis and thus energy expenditure, which over time would reduce adiposity and consequently correct metabolic abnormalities associated with obesity. Broadly speaking, manipulation of UCP1 can be classified into two distinct ways: (i) acute activation of existing UCP1 and (ii) induction of UCP1. Induction of UCP1 can take several forms, i.e., the expansion of existing BAT depots, producing more UCP1 per gram of BAT, or inducing UCP1 within WAT (browning). However, the significance of either activating UCP1 within classical BAT depots or browning WAT in humans in terms of energy expenditure remains unclear. Using available data on organ-specific metabolic rates, and our own data on (a) the metabolic rate of human BAT following acute cold exposure and (b) human WAT that has undergone browning, we present theoretical calculations to estimate the potential impact of UCP1 on energy expenditure in humans (Tables 1 and 2).

**Table 1:** **Impact of acute non-shivering cold exposure and severe burns on whole-body energy expenditure**.

	Normal^a^ (kcal/day)	Cold exposure (kcal/day)	Severe burns^d^ (kcal/day)
BAT	1 (0.1)	127 (7.0)^c^	191 (6.3)^e^
WAT	68 (4.0)^b^	68 (3.7)	205 (6.7)^f^
Muscle	368 (21.7)	368 (20.2)	748 (24.5)
Liver	362 (21.3)	362 (19.8)	684 (22.4)
Heart	146 (8.6)	146 (8.0)	368 (12)
Kidney	137 (8.1)	137 (7.5)	244 (8.0)
Brain	338 (19.9)	338 (18.5)	338 (11.1)
Other	277 (16.3)	277 (15.2)	277 (9.1)
Whole body	1697	1823	3053

*Values for each organ/tissue are presented as kilocalories/day with the percent contribution to whole-body energy expenditure in brackets*.

*^a^Values for thermoneutral conditions are taken from Rolfe and Brown (7)*.

*^b^Values for white adipose tissue (WAT) are taken from Gallagher et al. (48)*.

*^c^Values for cold exposure (5 h at ~18°) were derived from Chondronikola et al. (24), assuming a 7.5% increase in resting energy expenditure following acute cold exposure, which was attributable to brown adipose tissue (BAT) activation*.

*^d^Values for burn victims are taken from Wilmore and Aulick (49) for a patient with full-thickness burns encompassing 50% of their total body surface area, which results in an 80% increase in resting energy expenditure*.

*^e^As chronic cold exposure results in a 50% increase in BAT volume [van der Lans et al. (44)], BAT values derived from Chondronikola et al. (24) were multiplied by a factor of 1.5*.

*^f^WAT values for healthy individuals derived from Gallagher et al. (48) were multiplied by a factor of 3 to account for the increase in leak respiratory capacity of WAT seen in burn victims (47)*.

**Table 2:** **The contribution of various tissues to the increase in whole-body metabolic rate accompanying acute non-shivering cold exposure and severe burns**.

	Cold exposure		Severe burns	
	**Δ** kcal/day	% of increase	**Δ** kcal/day	% of increase
BAT	126	100	190	14
WAT	0	–	136	10
Muscle	0	–	379	28
Liver	0	–	322	24
Heart	0	–	222	16
Kidney	0	–	107	8
Brain	0	–	0	0
Other	0	–	0	0
Whole body	126	100	1356	100

*Values for acute cold exposure and severe burn injury reported in Table 1 are presented as the change (Δ) from healthy values and also as a percentage (%) of the increase in whole-body energy expenditure*.

Since UCP1 is inactive in the presence of inhibitory purine nucleotides such as ATP (50), BAT makes a negligible contribution to whole-body REE given that ~100 g of BAT represents ~0.1% of total body mass of a 70 kg individual. Similarly, in an individual with ~20% total fat mass, WAT accounts for a small (~4%) portion of whole-body REE (48) (see Table 1). Following acute (5 h) acclimation to mild cold (5 h at ~18°), our data (24) suggest that on average there is a 7.5% increase in REE. This acute intervention transforms BAT from being a quiescent tissue to one that makes a comparable contribution to whole-body REE as the heart or the kidneys (Table 1). In a 70 kg individual, this activation of BAT increases REE by 127 kcal (Table 1), which if maintained for 30 days would combust 0.5 kg of adipose tissue.

Burn trauma results in a significant increase in REE (51), which persists for months if not years post injury (52). While many ATP-consuming processes increase after burn, increased ATP turnover only explains 50–60% of burn-induced hypermetabolism (53). This means that thermogenesis represents a significant portion of hypermetabolism in burn victims, which makes sense since these patients have a compromised skin barrier and inability to thermoregulate. In order to estimate the potential contribution of BAT used burn injury as a model of severe adrenergic stress which is accompanied by hypermetabolism. We made calculations of whole-body and tissue-specific energy expenditure based on a patient with a burn covering 50% of their total body surface area, which resulted in an 80% increase in REE (49).

While the impact of burn trauma on BAT is largely unknown, we theorized that like chronic intermittent cold exposure, prolonged adrenergic stress following severe burn trauma would expand BAT depots. Assuming an ~50% increase in BAT volume as seen with 10 days of mild cold exposure (44), BAT would account for 191 kcal of total REE (Table 1). Here, while BAT makes a greater contribution to REE in absolute terms, since burn injury results in a large increase in REE, the relative contribution of BAT to whole-body REE is slightly lower (6.3%) when compared to cold exposure (7%) (Table 1). Perhaps more strikingly, when considering WATs contribution to REE in burn victims, the threefold increase in thermogenic capacity we found in subcutaneous WAT of burn victims would increase WATs contribution to whole-body REE to ~7% (Table 1). Thus, WAT and BAT may make similar absolute and relative contributions to whole-body REE in burn survivors. Again though, we should point out that these calculations are based on an individual with around 100 g of BAT and 15 kg of WAT, underscoring the profound difference in thermogenic potential per gram of tissue between BAT and WAT.

While the chronic adrenergic stress accompanying severe burn trauma provides a unique model where human WAT undergoes browning, it also offers significant insight into the physiological impact of achieving UCP1 activation and recruitment. In Table 2, we present the values reported in Table 1 as the change from normal and also as a percentage in which each tissue contributes to the increase in whole-body REE. While the 127 kcal increase in REE following acute cold exposure is attributable to BAT, several organs contribute to the massive (1230 kcal) increase in REE following burn severe trauma. For example, while burn trauma may result in large absolute increases in BAT and WAT metabolic rates, their relative contributions to total REE are less impressive. Indeed, skeletal muscle (28%), the liver (24%), and the heart (16%) contribute most to burn-induced hypermetabolism, followed by BAT (14%) and WAT (10%) (Table 2).

We make the point above to underscore the fact that while the chronic stress response to burns may augment BAT and WAT thermogenesis, the systemic milieu which brings these adaptations in adipose tissue energy expenditure likely impacts many other organs. While our data on burn victims (47) provide novel evidence that human WAT can indeed undergo browning under very severe and extended adrenergic stress, this extreme pathophysiological state has an impact on nearly every organ system in the body. Thus, with all the available evidence to date, we suggest that the therapeutic potential of UCP1-positive mitochondria in combating obesity and its metabolic complications will likely be realized in humans by the acute activation of existing BAT and/or the expansion of BAT depots. If WAT browning holds any therapeutic potential in the context of obesity, the development of safe tissue-specific agents to induce browning is needed before this potential can be realized.

## Summary

Adipocytes with an abundance of UCP1-positive mitochondria are unique in that gram for gram they have a respiratory capacity akin to that of muscle or liver, yet only minimal ability to produce ATP. The physiological role ascribed to this adaptation in mitochondrial energy transduction is heat production, facilitating thermoregulation in mammals. In the context of the global obesity epidemic, there is now much interest in the environmental and/or pharmacological exploitation of this thermoregulatory mechanism, since increased metabolic rate will combat obesity and the deleterious metabolic phenotype it incurs. In humans, a growing body of evidence suggests that acute activation of BAT can indeed have a meaningful impact on REE and intermediary glucose and lipid metabolism. However, whether brown adipocytes can be recruited in WAT and whether this alters REE or macronutrient metabolism remains unclear. Few human models mimic the extremity of confining rodents to near-freezing temperatures for several weeks, and in our study of severely burned patients, while prolonged stress does indeed brown WAT, several other organs are also significantly affected. Thus, to avoid the fate of the mitochondrial uncoupler dinitrophenol, future strategies aimed at augmenting UCP1 content and function in humans must strive to be tissue specific.

## Conflict of Interest Statement

The authors declare that the research was conducted in the absence of any commercial or financial relationships that could be construed as a potential conflict of interest.
